# Polyphenol-Rich Extracts from *Annurca* Apple Differentially Modulate Oxidative Stress-Induced Senescence in Human Dermal Fibroblasts

**DOI:** 10.3390/antiox15030372

**Published:** 2026-03-16

**Authors:** Claudia Moriello, Nicola Alessio, Pasquale Perrone, Tiziana Squillaro, Stefania D’Angelo, Umberto Galderisi, Giovanni Di Bernardo

**Affiliations:** 1 Department of Experimental Medicine, Luigi Vanvitelli Campania University, 80138 Naples, Italy; claudia.moriello@unicampania.it (C.M.); umberto.galderisi@unicampania.it (U.G.); gianni.dibernardo@unicampania.it (G.D.B.); 2 Department of Medical, Human Movement and Well-Being Sciences (DiSMMeB), Parthenope University of Naples, 80133 Naples, Italy; pasquale.perrone@collaboratore.uniparthenope.it (P.P.); stefania.dangelo@uniparthenope.it (S.D.); 3 Department of Life, Health and Healthcare Professions, UniLink, 00165 Rome, Italy; t.squillaro@unilink.it

**Keywords:** senescence, human dermal fibroblast, polyphenols, *Annurca* apple, oxidative stress, mitochondrial dysfunction, senotherapeutics, skin aging

## Abstract

Cellular senescence of dermal fibroblasts is a central mechanism underlying skin aging and is closely linked to oxidative imbalance and mitochondrial dysfunction. In this study, we investigated the senotherapeutic effects of polyphenol-rich extracts obtained from peel and flesh of *Malus pumila* Mill. cv. *Annurca* at different ripening stages. Senescence was induced in human dermal fibroblasts by oxidative stress, and the biological activity of unripe and ripe apple extracts was comparatively evaluated. The effects of the treatments were assessed by analyzing cell viability, apoptotic response, oxidative stress levels, mitochondrial functionality, and established molecular and functional markers of cellular senescence. All extracts were well tolerated by young fibroblasts and selectively promoted apoptosis in senescent cells. However, marked differences in biological activity were observed depending on fruit tissue and ripening stage. Unripe peel extract consistently reduced senescence-associated markers, attenuated oxidative stress, and restored mitochondrial homeostasis, indicating a combined senomorphic and senolytic activity. In contrast, ripe peel extract displayed a predominantly senolytic profile associated with increased oxidative stress, while flesh-derived extracts exerted weaker or incomplete effects on senescence pathways. These results demonstrate that *Annurca* apple polyphenols modulate cellular senescence depending on fruit part and ripening stage. Peel extracts, particularly from unripe fruits, represent potent and sustainable natural senotherapeutics, highlighting the potential of agro-food by-products for anti-aging and skin rejuvenation applications.

## 1. Introduction

Cellular senescence is a state of permanent cell cycle arrest that limits the proliferative lifespan of diploid cells [1]. This phenomenon was first described in the 1960s by Hayflick and Moorhead, who observed that human diploid fibroblasts in culture undergo a finite number of divisions before irreversibly ceasing proliferation [2]. Beyond its historical definition, cellular senescence is now recognized as a physiological process essential for normal development, tissue homeostasis, remodeling, and repair [3]. Also, it serves as a critical tumor-suppressive mechanism by preventing the expansion of damaged or dysfunctional cells.

However, when aberrantly activated or chronically sustained, senescence becomes a key hallmark of aging and a major contributor to age-related pathologies [4]. Senescent cells are characterized by profound functional and metabolic alterations, including persistent activation of DNA damage responses, dysregulation of cell cycle checkpoints, and increased expression of cyclin-dependent kinase inhibitors such as p21 and p16 [1,5]. In addition, senescent cells develop a distinctive secretory phenotype and exhibit marked changes in redox balance and mitochondrial function [6]. In the skin, dermal fibroblasts play a pivotal role in maintaining skin integrity through regulation of extracellular matrix composition, wound healing, and tissue homeostasis [7]. Age-associated dysfunction of dermal fibroblasts leads to structural deterioration, persistent inflammation, and impaired regenerative capacity, ultimately contributing to skin aging [8].

Both intrinsic (chronological) and extrinsic aging driven by environmental insults, such as ultraviolet radiation, air pollution, smoking, and poor nutrition, converge on the induction of cellular senescence in dermal fibroblasts and epidermal keratinocytes, thereby accelerating skin aging [9]. At the molecular level, mitochondria emerge as central regulators of this process, acting as both major sources and primary targets of reactive oxygen species (ROS) [10]. Excessive mitochondrial ROS production promotes oxidative stress, mitochondrial dysfunction, and cumulative cellular damage, reinforcing senescence-associated phenotypes and tissue degeneration [11].

In recent years, a new class of molecules called senotherapeutics has emerged, with the aim of selectively eliminating or modulating the phenotype of senescent cells [12].

Senotherapeutic compounds can act either as senolytics, inducing apoptosis preferentially in senescent cells, or as senomorphics, attenuating senescence-associated features without triggering cell death [13,14,15]. Importantly, emerging evidence indicates that effective senotherapeutic activity is not necessarily associated with antioxidant properties alone and may instead rely on the ability to exploit the intrinsic metabolic and redox vulnerabilities of senescent cells [16,17].

Within this framework, the research focused on the study of natural molecules with senotherapeutic properties. Recent evidence has highlighted the potential of polyphenol-rich natural extracts, including those derived from *Phyllanthus indofischeri* (Giant Indian Gooseberry, GIG) and *Helichrysum italicum*, in counteracting skin aging by modulating cellular senescence, promoting cutaneous regeneration, and enhancing antioxidant and pro-collagen activities in human skin cells [18,19].

Recent studies have shown that the natural compounds present in *Annurca* apples, (*Malus pumila* Mill. cv. *Annurca*), a traditional cultivar native to Campania (Italy), have several beneficial effects on human health [20,21].

Extracts from this fruit are remarkably rich in polyphenolic compounds, including quercetin, catechin, epicatechin, chlorogenic acid, and procyanidins [22]. Notably, the qualitative and quantitative composition of apple polyphenols varies substantially depending on both the anatomical part of the fruit and the stage of ripening, with apple peel and unripe fruits generally displaying higher concentrations of oligomeric polyphenols. Several of these polyphenolic constituents have recently been recognized in the literature as senolytic agents [23,24,25,26].

On this basis, the present study was designed to systematically investigate the senotherapeutic effects of polyphenol-rich extracts derived from different anatomical parts (peel and flesh) and ripening stages (unripe and ripe) of *Annurca* apple. We use an in vitro model of acute H_2_O_2_-induced senescence in human dermal fibroblasts (HDFs). By directly comparing four distinct preparations, unripe flesh (URF), unripe peel (URP), ripe flesh (RF), and ripe peel (RP), we evaluated their ability to modulate key hallmarks of cellular senescence, including senescence-associated β-galactosidase activity and the expression of RB1, p53, p21, and p16. In parallel, we assessed oxidative stress levels and mitochondrial integrity, with particular emphasis on mitochondrial membrane potential, dynamics, and biogenesis, to delineate the mechanistic link between mitochondrial homeostasis and the senescent phenotype. Overall, the results of this work are expected to advance our understanding of mitochondria-centered senotherapeutic mechanisms and to foster the development of sustainable, polyphenol-based interventions for skin rejuvenation and healthy aging.

## 2. Materials and Methods

### 2.1. Fruit Collection

*Annurca* apples (*Malus pumila* Mill. cv. *Annurca*) were harvested in 2024 from an orchard located in Giugliano in Campania (Naples, Italy). Fruits were collected from multiple trees within the same cultivation area to ensure representative sampling. Only apples with uniform size, absence of visible defects, and homogeneous physiological maturity were selected. The fruits were harvested in September in the pre-climacteric phase, characterized by green skin and incomplete ripeness. Some of these unripe apples were at once processed for analytical purposes. The remaining fruits underwent the traditional post-harvest reddening process in “melai”, which consist of a raised bed of well-drained soil covered with a layer of straw, where the apples were exposed to natural sunlight for about a month. After the reddening phase, samples of ripe fruits were collected and processed for comparative analysis [22].

### 2.2. Polyphenol Extraction

Prior to extraction, apple peel was manually separated from the flesh, and the seed cores were completely removed. The resulting tissues were then processed independently. *Annurca* apple samples were homogenized for 10 min using a Tefal Rondo 500 homogenizer (Tefal, Rumilly, France) in the presence of an extraction solvent mixture (1:1 *w*/*v* ratio of apple to solvent) consisting of 80% methanol and 20% water, supplemented with 0.18 N HCl. Following centrifugation at 18,000× *g* for 30 min, the resulting supernatant was dried under vacuum using the Eppendorf Concentrator Plus (Eppendorf AG, Hamburg, Germany). The dried residues were reconstituted in PBS and stored at −80 °C until analysis. Total polyphenol content was determined using the Folin–Ciocalteu phenolic assay (Sigma-Aldrich, St. Louis, MO, USA). Briefly, 10 μL of each extract was combined with 5 mL of Folin–Ciocalteu reagent, 4 mL of Na_2_CO_3_ (7.5% *w*/*v*), and 990 μL of deionized water. After incubation at room temperature for 2 h, absorbance was measured at 765 nm using a UV-3100PC spectrophotometer (VWR International, Radnor, PA, USA). Quantification was performed using a catechin calibration curve, and polyphenol content was expressed as millimolar (mM) concentrations, calculated as millimoles of polyphenols (CAEq) per liter of extract. All extract concentrations are expressed as catechin equivalents to allow comparison among samples despite differences in polyphenolic composition [22].

### 2.3. Cell Culture

Human dermal fibroblasts were obtained from the American Type Culture Collection (ATCC, Manassas, VA, USA) and grown in low-glucose (1 g/L) DMEM containing 10% Fetal Bovine Serum, 4 mM L-glutamine, and 100 U/mL penicillin–streptomycin. All cell cultures were maintained at 37 °C with 5% CO_2_ in a humidified environment.

### 2.4. Induction of Acute Senescence and Extract Treatment

Human dermal fibroblasts were treated with hydrogen peroxide. Cells were incubated in complete medium containing 300 μM H_2_O_2_ for 30 min [27]. After incubation, the medium was discarded and replaced with fresh medium, and the cells were further incubated for 72 h before addition of the extract. Treatment with the extract was performed using a concentration of URF (10 µM), URP (50 µM), RF (50 µM), and RP (100 µM) for either 24 or 48 h depending on the specific assay.

### 2.5. In Situ Senescence-Associated β-Galactosidase Assay

Senescent cells were detected using the Senescence SA-β-galactosidase Staining Kit (Cell Signaling Technology, Danvers, MA, USA) according to the manufacturer’s procedure. Cells were fixed and stained, and blue-stained cells were counted in at least five different fields using a bright-field microscope. The results are reported as a percentage of positive cells compared to total nuclei [28].

### 2.6. Cell Viability Assay

Cell Counting Kit-8 (Dojindo EU GmbH, Munchen, Germany) was used to determine the cytotoxicity of the different extracts on HDF cells, according to the manufacturer’s procedure. We seeded cells at a concentration of 5000 cells/well in 100 μL culture medium containing various amounts of extracts (final concentration 1 up to 500 or 1000 μM) into 96 wells microplates and incubated cell cultures for 48 h. After the incubation period, CCK8 assay was performed and viability was detected using GloMax^®^ Discover microplate reader (Promega, Milan, Italy) at 450 nm wavelength.

### 2.7. Apoptosis Detection

Apoptosis was evaluated by flow cytometry using the Annexin V-FITC/PI Apoptosis Kit (E-CK-A211, Elabscience, Houston, TX, USA). The cells were collected, washed, and dyed according to the manufacturer’s instructions. The early and late apoptotic populations were quantified using a BD Accuri C6 cytometer (Becton, Dickinson and Company, Franklin Lakes, NJ, USA).

### 2.8. Mitochondrial Membrane Potential (JC-1)

Mitochondrial integrity was measured using the JC-1 Mitochondrial Membrane Potential Assay Kit (Thermo Fisher, Waltham, MA, USA). JC-1 dye was used to label cells, which were then examined using flow cytometry. The red/green fluorescence ratio was used as a sign of mitochondrial polarization.

### 2.9. Reactive Oxygen Species Measurement (DCFH-DA Assay)

ROS production was measured by the conversion of fluorogenic 2,7dichlorodihydrofluorescein diacetate (DCFH-DA) to highly fluorescent dichlorofluorescein diacetate within cells by ROS. Cells were treated with extracts and 10 µM DCFH-DA. Fluorescence (excitation 485 nm, emission 535 nm) was continuously monitored for 48 h at 37 °C using a CO_2_-independent medium in a GloMax^®^ Discover microplate reader (Promega, Milan, Italy).

### 2.10. Western Blotting

Total proteins were extracted using Lysis buffer supplemented with protease and phosphatase inhibitor. The protein lysis buffer was prepared to obtain the following final concentrations: 50 mM Tris-HCl (pH 7.4), 250 mM NaCl, 0.1% (*v*/*v*) Triton X-100, 50 mM NaF, 4 mM PMSF, as well as phosphatase inhibitor (P0001, Sigma-Aldrich, Saint Louis, MO, USA) and protease inhibitor (G135, ABM, Richmond, BC, Canada) (used according to the manufacturer’s recommended final concentration). They are incubated for 30 min at 4 °C and centrifuged 13,000× *g* for 10 min. The supernatants were transferred to tubes and quantified by using Bio-Rad Protein Assay Dye Reagent Concentrate (cat# 5000006, Bio-Rad, Milan, Italy). Protein samples (10 μg total protein) were heated at 95 °C for 5 min in Laemmli 2x buffer (Sigma-Aldrich, Saint Louis, MO, USA). Proteins were separated by SDS-PAGE and transferred to PVDF membranes. Membranes were incubated overnight at 4 °C with the following primary antibodies: RB (mw 105 kD) (#9309, CellSignaling, Danvers, MA, USA), P53 (mw 53 kD) (sc-126, SantaCruz Biotechnology, Dallas, TX, USA), P21 (mw 21 kD) (sc-6246, SantaCruz Biotechnology, Dallas, TX, USA), P16 (mw 16 kD and 32 kD the omodimer) (E-AB-65673, Elabscience, Houston, TX, USA), MFN1 (mw 83 kD) (E-AB-93135, Elabscience, Houston, TX, USA), MFN2 (mw 86 kD) (E-AB-32025, Elabscience, Houston, TX, USA), FIS1 (mw 16 kD) (E-AB-67035, Elabscience, Houston, TX, USA), DRP1 (mw 82 kD) (E-AB.-93308, Elabscience, Houston, TX, USA), MitoBiogenesis™ Western Blot Cocktail (ab123545, Abcam, Cambridge, UK), beta Actin (mw 42 kD) (sc-47778, SantaCruz Biotechnology, Dallas, TX, USA), followed by HRP-conjugated secondary antibodies (Elabscience, Houston, TX, USA). Detection was performed using ECL Plus reagent (Elabscience, Houston, TX, USA) and imaged with an Azure 300 system (Azure Biosystems, Dublin, CA, USA). Western blot bands were quantified by densitometric analysis using Image Lab software 6.1 (Bio-Rad, Milan, Italy). Raw Western blot data are in Supplementary File S1.

### 2.11. Statistical Analysis

Every experiment was carried out in triplicate. The data are presented as mean ± SEM. Statistical significance was established using one-way ANOVA and Tukey’s post hoc test in GraphPad Prism 10 (GraphPad Software, La Jolla, CA, US). A *p*-value of < 0.05 was considered statistically significant.

## 3. Results

### 3.1. Differential Polyphenolic Composition of the Extracts

**Figure 1:**
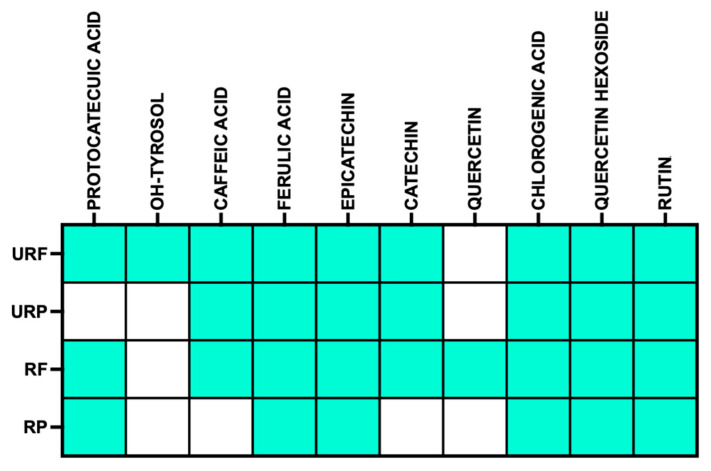
**Binary heat map of polyphenolic compound presence.** Binary heat map showing the presence (light blue) or absence (white) of selected polyphenolic compounds across the analyzed samples.

In a previous study, the qualitative polyphenolic profile of the analyzed samples was characterized, leading to the identification of several polyphenolic compounds. The presence or absence of these compounds across the different samples is summarized in Figure 1, which provides an overview of the polyphenol distribution and highlights qualitative differences among the extracts [22].

### 3.2. Citotoxicity Evaluation and Apoptosis Induction

**Figure 2:**
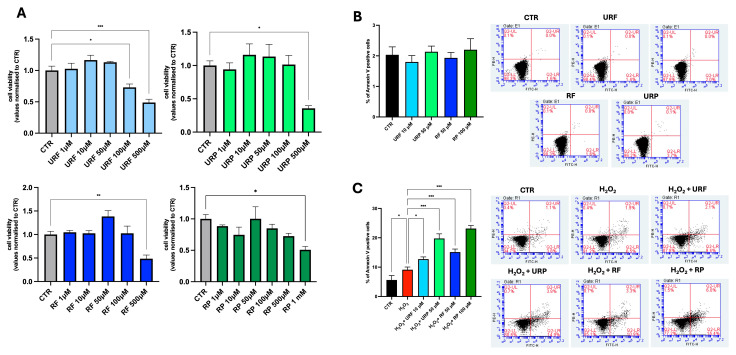
**Effects of apple extracts on HDF viability and apoptosis.** (**A**) CCK-8 assay showing cell viability at 48 h after treatment with URF, URP, RF, and RP. (**B**,**C**) Annexin V flow cytometry analysis performed at 24 h in young (**B**) and senescent (**C**) HDFs treated with different apple extracts. All experiments were performed in three independent biological replicates. Data are expressed as mean ± SEM. Statistical analysis was conducted using one-way ANOVA followed by Tukey’s post hoc test. Statistical significance is indicated by asterisks as follows: *p* < 0.05 (*), p < 0.01 (**), and p < 0.001 (***).

The first step of our study was to evaluate the cytotoxicity of apple extracts on young human dermal fibroblasts. This approach allowed us to identify concentrations tenfold lower than the cytotoxic threshold to be used in subsequent experiments. Specifically, concentrations ranging from 1 µM to 500/1000 µM were tested, and the data shown in Figure 2A indicate that the selected working concentrations were 10 µM for URF, 50 µM for URP and RF, and 100 µM for RP.

Next, we assessed whether the extracts induced apoptosis in young HDFs. As shown in Figure 2B, none of the extracts caused a significant increase in the percentage of apoptotic cells compared with the control. Importantly, the absence of apoptosis induction in young HDFs indicates that the selected concentrations of all extracts are not associated with non-specific cytotoxic effects.

Subsequently, the senolytic potential of the extracts was evaluated following acute induction of senescence using hydrogen peroxide (H_2_O_2_). Specifically, cells were exposed to 300 μM H_2_O_2_ for 30 min, followed by a 72 h recovery period to allow establishment of the senescent phenotype. Only after this phase were the extracts administered to already adherent cells Notably, all extracts resulted in an increase in the percentage of apoptotic cells under these conditions. In particular, Figure 2C shows a modest increase compared with the H_2_O_2_-treated control in cells treated with URF and RF, whereas a markedly stronger effect was observed in samples treated with URP and RP, with approximately a twofold increase in apoptosis. This selective pro-apoptotic effect in senescent but not young fibroblasts supports a senolytic rather than a general cytotoxic activity of the extracts.

### 3.3. Regulation of Oxidative Stress and Mitochondrial Remodeling

**Figure 3:**
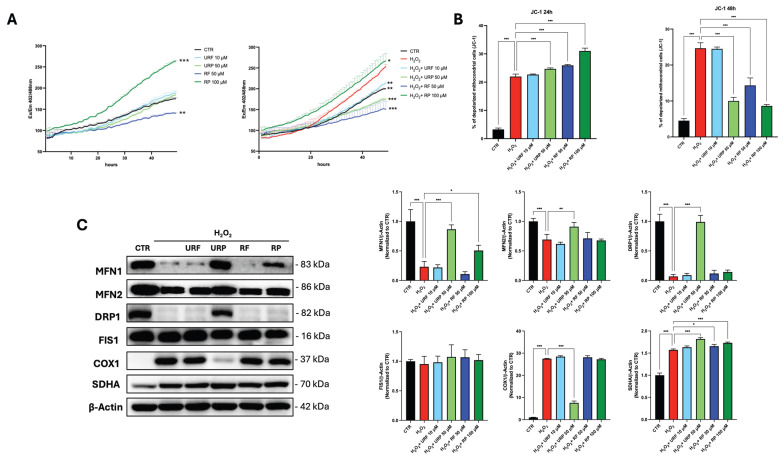
**Effects of apple extracts on oxidative stress, mitochondrial function, and dynamics in HDFs.** (**A**) DCFH-DA assay showing intracellular ROS levels following 48 h treatment with URF, URP, RF, and RP in young and senescent HDFs (Statistical significance is indicated vs. control in the first graph and vs. H_2_O_2_-treated control in the second graph.) (**B**) JC-1 assay evaluating changes in mitochondrial membrane potential at 24 and 48 h. (**C**) Protein expression analysis of mitochondrial fusion and fission markers (MFN1, MFN2, DRP1, and FIS1) and mitochondrial biogenesis markers (COX1 and SDHA) in senescent HDFs treated with different apple extracts. The graphs show the quantification of Western blot bands performed by using β-Actin as the loading control. All experiments were performed in three independent biological replicates. Data are presented as mean ± SEM. Statistical analysis was carried out using one-way ANOVA followed by Tukey’s post hoc test. Statistical significance is indicated by asterisks as follows: *p* < 0.05 (*), *p <* 0.01 (**), and *p <* 0.001 (***). Raw Western blot data are depicted in Supplementary File S1.

ROS levels were monitored using DCFH-DA fluorescence assay over 48 h (Figure 3A). Control cells maintained basal ROS levels. Treatment with H_2_O_2_ induced a marked elevation in ROS accumulation, reflecting severe oxidative stress. Treatment with URF, URP and RF effectively attenuated ROS generation. In contrast, RP exhibited prooxidant behavior, showing elevated ROS accumulation. This divergent redox behavior is likely attributable to differences in the molecular composition of the extracts, as synergistic interactions among polyphenols can markedly influence their overall biological activity. In particular, variations in the relative abundance and chemical form of individual polyphenols may determine whether the net redox effect of a polyphenol mixture is antioxidant or prooxidant [29].

We next investigated mitochondrial status by assessing mitochondrial membrane depolarization using the JC-1 assay and mitochondrial dynamics by Western blot analysis. As shown in Figure 3B, a 24 h treatment revealed an increase in mitochondrial membrane depolarization in H_2_O_2_ treated cells compared with untreated control cells. A further enhancement of mitochondrial depolarization was observed following treatment with URP, RF, and RP, whereas no significant alterations were detected in cells treated with URF.

This early effect was transient, as analysis at 48 h showed a reduction in the percentage of cells displaying depolarized mitochondria in the URP-, RF-, and RP-treated groups, suggesting a restoration of physiological mitochondrial function. Consistently, URF-treated cells did not exhibit any significant changes compared with the senescent control.

Mitochondrial dynamics were subsequently evaluated (Figure 3C) by analyzing the expression of MFN1 and MFN2, two key regulators of mitochondrial fusion. The data show that H_2_O_2_ treatment induced a marked reduction in the expression of both proteins.

Notably, treatment with URP restored MFN1 and MFN2 expressions to levels comparable to those of the untreated control. In contrast, RP treatment resulted in only a partial recovery of MFN1 expression, while no restoration of either fusion protein was observed in the remaining treatment groups.

Proteins involved in mitochondrial fission, DRP1 and FIS1, were also analyzed. No significant changes in FIS1 expression were detected across any of the experimental conditions. In contrast, DRP1 expression was strongly reduced following H_2_O_2_ treatment and was restored exclusively in cells treated with URP.

Finally, mitochondrial biogenesis was investigated by assessing the expression of SDHA and COX1. H_2_O_2_ treatment led to an increase in COX1 expression, which was reduced exclusively by URP treatment. However, this increase in SDHA expression did not uniformly correlate with improved mitochondrial function, suggesting that enhanced biogenesis alone is insufficient to restore mitochondrial homeostasis in senescent cells.

### 3.4. Modulation of Cellular Senescence Hallmarks

**Figure 4:**
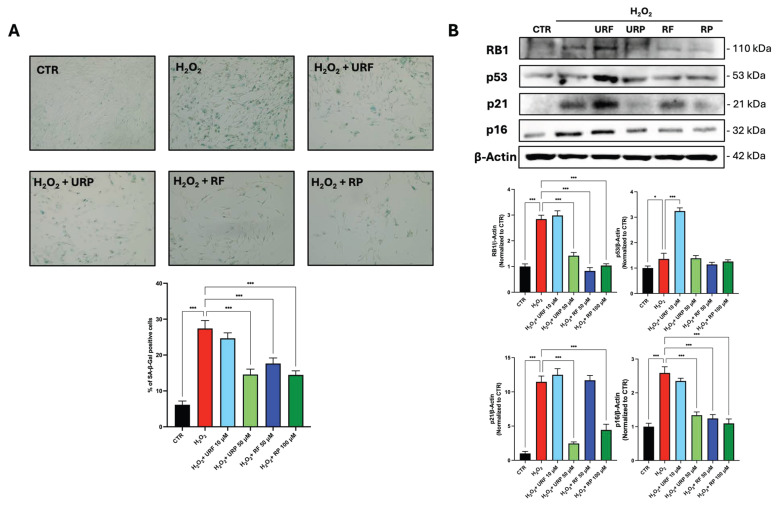
**Effects of apple extracts on cellular senescence markers.** (**A**) Percentage of β-galactosidase-positive cells as determined by SA-β-gal staining. (**B**) Protein expression levels of senescence-related markers (RB1, p53, p21, and p16) following treatment with apple extracts. The graphs show the quantification of Western blot bands performed by using β-Actin as the loading control. All experiments were performed in three independent biological replicates. Data are presented as mean ± SD. Statistical analysis was carried out using one-way ANOVA followed by Tukey’s post hoc test. Statistical significance is indicated by asterisks as follows: *p* < 0.05 (*) and *p <* 0.001 (***). Raw Western blot data and raw microscope images are depicted in Supplementary File S1.

Cellular senescence was further evaluated by assessing senescence-associated β-galactosidase (SA-β-gal) assay and the expression of key senescence-related proteins (Figure 4). As shown in Figure 4A, H_2_O_2_ treatment markedly increased the percentage of SA-β-gal-positive cells compared with untreated controls, confirming the induction of a senescent phenotype.

Treatment with URP, RF, and RP resulted in a marked reduction in the percentage of SA-β-gal-positive cells, with URP and RP showing the most pronounced effects, whereas URF treatment did not produce a significant change compared with H_2_O_2_-treated cells.

Consistently, analysis of senescence-associated proteins revealed that H_2_O_2_ exposure induced an increase in the expression levels of RB1, p53, p21 and p16 (Figure 4B). Treatment with URP and RP significantly reduced the expression of RB1, p21, and p16, restoring levels closer to those observed in control cells. In RF-treated cells, a reduction in RB1 and p16 expression was observed; however, p21 levels remained elevated. In contrast, URF treatment did not result in a decrease in senescence markers and was associated with a further increase in p53 protein levels, indicating that antioxidant activity alone is insufficient to counteract the senescent phenotype. Overall, among all tested extracts, URP consistently displayed the most coherent senotherapeutic profile, combining selective senolytic activity with restoration of mitochondrial homeostasis.

## 4. Discussion

In this study, we provide a comprehensive evaluation of the senotherapeutic properties of polyphenol-rich extracts obtained from different anatomical parts (peel and flesh) and ripening stages (unripe and ripe) of *Malus pumila* Mill. cv. *Annurca*, using an in vitro model of oxidative stress-induced premature senescence in human dermal fibroblasts. By integrating cellular viability, apoptotic response, oxidative stress, mitochondrial function and canonical senescence markers, our data reveal a marked functional heterogeneity among the extracts, highlighting unripe peel (URP) displaying a combined senolytic and senomorphic activity.

The induction of senescence by acute H_2_O_2_ exposure recapitulated key features of stress-induced premature senescence, including increased ROS accumulation, mitochondrial dysfunction, altered mitochondrial dynamics and activation of the p53/p21 and p16/RB pathways [30]. This model closely mirrors oxidative stress-driven fibroblast senescence observed during both intrinsic aging and environmentally induced skin aging [8,9]. Importantly, none of the apple extracts exhibited cytotoxic or pro-apoptotic effects in young fibroblasts at the selected working concentrations, indicating a favorable safety profile and supporting their suitability for senotherapeutic applications.

Polyphenols exhibit hormetic properties, acting as antioxidants at low concentrations and becoming prooxidant at higher concentrations due to the formation of unstable semiquinones [31]. In this study, all extracts were tested at concentrations tenfold lower than those found to be cytotoxic in young cells, a range commonly associated with prooxidant activity. Consistent with this paradigm, RP displayed a prooxidant profile under our experimental conditions, whereas URF, URP, and RF exerted clear antioxidant effects, resulting in a reduction in intracellular ROS levels [32].

In complex polyphenol mixtures, however, a net increase in ROS is not necessarily observed, as individual compounds differ in their concentration thresholds for the antioxidant–prooxidant switch [33]. In addition, polyphenolic mixtures may include compounds capable of stabilizing or reducing the quinone and semiquinone forms generated by the oxidation of other polyphenols, thereby limiting the accumulation of reactive intermediates and preserving an overall antioxidant behavior even at relatively high concentrations [34].

In this context, the presence of caffeic acid and catechin in URF, URP, and RF appears to prevent ROS accumulation, likely by exerting a redox-buffering effect through the stabilization or reduction in oxidized polyphenols [35].

Studies have shown that when different phenolic compounds are mixed, their combined antioxidant/prooxidant activities are not simply additive [29]. Instead, synergistic, antagonistic, or neutral interactions can emerge depending on the specific compounds and ratios involved. It is therefore reasonable to hypothesize that, within a complex mixture, the prooxidant activity of one polyphenol may be neutralized by another polyphenol with stronger iron-chelating capacity and/or greater semiquinone stability [36,37].

Despite these distinct redox profiles, all extracts induced an increase in apoptosis in senescent cells. This observation suggests that apoptotic induction is not directly driven by ROS modulation alone. Rather, the consistent alterations in mitochondrial membrane polarization observed across treatments point to mitochondrial depolarization as a key upstream event underlying apoptosis. Thus, irrespective of their antioxidant or prooxidant behavior, the extracts appear to converge on mitochondrial dysfunction, ultimately triggering mitochondria-dependent apoptotic pathways in senescent cells [4].

Mitochondria represent both a major source and a critical target of oxidative stress during senescence. In line with previous reports, H_2_O_2_-treated fibroblasts exhibited mitochondrial membrane depolarization and dysregulation of mitochondrial dynamics, reinforcing the concept of mitochondria-driven senescence [11,38]. Interestingly, URP, RF, and RP induced an early increase in mitochondrial depolarization at 24 h, followed by a significant reduction at 48 h. This biphasic response may reflect, at least in part, an initial mitochondrial stress that triggers adaptive remodeling, ultimately restoring mitochondrial function. Such transient mitochondrial perturbations have been described as beneficial hormetic signals promoting mitochondrial quality control and cellular adaptation [39].

Although the reduction in ROS in URP-treated samples, together with mitochondrial depolarization at 24 h, may seem contradictory, mitochondrial ROS production is not linearly related to membrane potential (Δψm). Excessive ROS generation is often associated with mitochondrial hyperpolarization, which promotes electron backpressure within the electron transport chain. A moderate depolarization can instead relieve this pressure, reducing electron leakage and consequently lowering ROS production.

In addition, polyphenols at specific concentrations may exert mild uncoupling effects, dissipating Δψm while limiting mitochondrial ROS generation. Senescent cells are frequently characterized by altered mitochondrial function and persistent hyperpolarization; therefore, forced depolarization may represent a mechanism through which URP modulates mitochondrial dysfunction.

Taken together, the observed decrease in ROS concomitant with mitochondrial depolarization can be interpreted as mechanistically coherent rather than contradictory.

In contrast, URF did not significantly affect mitochondrial membrane potential at either time point, suggesting a limited capacity to engage mitochondrial stress-response pathways. This lack of mitochondrial remodeling likely contributes to the inability of URF to attenuate senescence markers, despite its antioxidant properties. Notably, URF treatment was associated with a further increase in p53 expression, indicating the activation of a DNA damage or stress-response pathway rather than senescence mitigation. This finding underscores the complexity of polyphenol–cell interactions and suggests that certain polyphenolic profiles may reinforce stress signaling rather than promote senomorphic or senolytic effects [40].

The analysis of mitochondrial dynamics further delineated the unique efficacy of URP. H_2_O_2_-induced senescence was characterized by a marked downregulation of the fusion proteins MFN1 and MFN2, consistent with impaired mitochondrial networking in senescent cells [39]. URP treatment fully restored the expression of both MFN1 and MFN2, as well as DRP1, indicating a coordinated reactivation of mitochondrial fusion–fission balance. Restoration of mitochondrial dynamics is increasingly recognized as a prerequisite for senescence attenuation and metabolic recovery [19]. RP partially restored MFN1 but failed to normalize MFN2 and DRP1, while RF and URF showed no significant effects, further emphasizing the superior mitochondrial-targeting capacity of URP.

Mitochondrial biogenesis markers provided additional insight into the metabolic remodeling induced by apple extracts [41]. Senescent cells exhibited increased expression of COX1 and SDHA, likely reflecting compensatory mitochondrial mass expansion in response to functional impairment, a phenomenon described as mitochondrial hyperfunction in senescence [42]. URP uniquely reduced COX1 expression, suggesting normalization of mitochondrial respiratory chain activity, whereas all extracts further increased SDHA levels. This divergence may reflect selective modulation of electron transport chain components and highlights the complexity of mitochondrial biogenesis regulation in senescent cells, although further functional analyses will be required to clarify this aspect.

At the functional level, modulation of mitochondrial homeostasis translated into differential regulation of senescence hallmarks. URP, RF, and RP significantly reduced SA-β-gal-positive cells, whereas URF failed to do so. Consistently, URP and RP markedly downregulated RB1, p21, and p16, key mediators of irreversible cell cycle arrest. RF partially reduced RB1 and p16 but did not affect p21, indicating an incomplete attenuation of the senescent program. These results suggest that RF may exert mild senomorphic effects, possibly by dampening specific senescence pathways without fully reversing growth arrest. Similar partial effects have been reported for other polyphenol-rich extracts with limited bioactive diversity [20].

RP displayed a distinct biological profile characterized by prooxidant behavior coupled with potent senolytic activity, consistent with the apoptosis data shown in Figure 2C. This behavior aligns with our previous demonstration that hormetic ROS increase can selectively eliminate senescent cells [22,26]. Strikingly, URP, URF, and RF also exhibited senolytic activity despite their antioxidant properties, suggesting that senolysis can be achieved through multiple convergent pathways. We hypothesize that mitochondrial membrane depolarization, observed consistently across all treatments at 24 h (Figure 3B), represents a common execution mechanism that operates independently of net ROS modulation. Thus, while RP exploits the ROS vulnerability of senescent cells, the other extracts likely trigger apoptosis through direct mitochondrial perturbation, highlighting the mechanistic diversity of polyphenol-induced senolysis.

This behavior is consistent with a senolytic mechanism, whereby increased oxidative stress selectively compromises the survival of senescent cells, which are known to exhibit heightened vulnerability to ROS [24,25]. Polyphenols such as quercetin and procyanidins, enriched in apple peel and particularly abundant in ripe tissues, have been previously identified as natural senolytics capable of selectively eliminating senescent cells [23,26].

The marked differences observed between peel- and flesh-derived extracts, as well as between unripe and ripe stages, likely reflect profound changes in polyphenolic composition during fruit development. Unripe peels are particularly enriched in oligomeric procyanidins and catechins, compounds with strong antioxidant, mitochondrial-protective, and senomorphic properties. In contrast, ripening is associated with increased accumulation of flavonols and oxidized polyphenols, which may shift biological activity toward prooxidant and senolytic effects [22].

As widely documented in the literature, natural senolytics of plant origin have attracted increasing scientific interest over the past decade [43].

In the context of skin aging, several botanical preparations have demonstrated senotherapeutic activity in human dermal fibroblasts. For instance, a standardized extract of *Solidago virgaurea* subsp. *alpestris* exhibited moderate senolytic effects and reduced SASP expression in stress-induced premature senescent fibroblasts [44]. Similarly, an extract from *Angelica acutiloba* roots led to the identification of ligustilide as a natural senolytic capable of selectively inducing apoptosis in senescent fibroblasts and improving skin structure in vivo [45]. Additional plant-derived preparations, such as Alpine rose leaf extract, have also been reported to decrease the proportion of senescent fibroblasts without affecting healthy cells, further supporting the feasibility of targeting cutaneous senescence through complex phytochemical mixtures [46].

In conclusion, our data identify *Annurca* apple polyphenols as potent modulators of cellular senescence in human dermal fibroblasts, with unripe peel extract exhibiting a robust senotherapeutic profile with both senomorphic and senolytic features through coordinated regulation of oxidative stress, mitochondrial function, and senescence-associated pathways. Ripe peel extract, in contrast, preferentially acts as a senolytic agent, while flesh-derived extracts display weaker or incomplete activity. These findings support the concept that fruit anatomical origin and ripening stage critically determine senotherapeutic efficacy and highlight *Annurca* apple extracts as promising natural candidates for anti-aging and skin rejuvenation strategies. Beyond their biological efficacy, the exploitation of apple peel, traditionally considered an agro-food waste, underscores the potential of by-product valorization as a sustainable source of high-value senotherapeutic compounds, supporting a circular economy-driven approach to skin aging intervention. Future studies in chronic and replicative senescence models, as well as in 3D skin equivalents, will be essential to further explore their translational potential.

### Limitations of the Study

The present investigation has several limitations that should be acknowledged. First, the polyphenolic extracts represent complex mixtures, and while qualitative composition was determined (Figure 1), the quantitative contribution of individual compounds to the observed biological effects remains unknown. This precludes definitive attribution of senotherapeutic activity to specific polyphenols. Second, although we demonstrate senolytic efficacy for all extracts, the precise molecular mechanisms were not fully elucidated. Our previous studies identified hormetic ROS increase as a central execution pathway for terpene- and stilbene-induced senolysis [32,47]. However, the current findings reveal that antioxidant extracts (URF, URP, RF) can also induce selective apoptosis, suggesting the existence of alternative mechanisms involving direct mitochondrial targeting. Future studies with purified individual polyphenols will be necessary to dissect these pathways. Third, the study was conducted in a single in vitro model of acute oxidative stress-induced senescence. Validation in chronic senescence models, replicative senescence, and organotypic skin cultures will be essential to confirm the translational relevance of these findings. Despite these limitations, this study provides a valuable preliminary characterization of *Annurca* apple by-products as sustainable sources of natural senotherapeutics.

## Data Availability

Data is contained within the article and Supplementary Material.

## Supplementary Materials

The following supporting information can be downloaded at https://www.mdpi.com/article/10.3390/antiox15030372/s1. Supplementary File S1 provides the raw and uncropped images of all Western blot experiments presented in this study.

## Abbreviations

The following abbreviations are used in this manuscript:

ROS	Reactive oxygen species
GIG	Giant Indian Gooseberry
HDFs	Human dermal fibroblasts
URF	Unripe flesh
URP	Unripe peel
RF	Ripe flesh
RP	Ripe peel
DCFH-DA	2,7dichlorodihydrofluorescein diacetate
SA-β-gal	Senescence-associated β-galactosidase
