# Supplementary material for: Polyphenol-Rich Extracts from Annurca Apple Differentially Modulate Oxidative Stress-Induced Senescence in Human Dermal Fibroblasts
Source: Antioxidants (Basel). 2026 Mar 16;15(3):372. doi: 10.3390/antiox15030372 (PMC13024385; doi:10.3390/antiox15030372)

Note:

- The red and blue fluorescent signals observed in the ladder image are instrument-related acquisition artifacts generated by the Azure imaging system during detection and do not represent sample-derived signals.

- Please note that image acquisition with the Azure image system allows for the adjustment of background noise using the so-called “gamma exposure” option. These adjustments affected only the display settings (LUT) and did not modify the raw 16-bit image data. This is what can be seen in the image on the right.

- The ladder signal is then acquired in bright field. In this condition, all the noise eliminated with the “gamma exposure” option is lost (left image).

- Please note that quantification does not depend on image modifications made with the “gamma exposure” option.

MFN1 ≈ mw 83 kDa fig 3

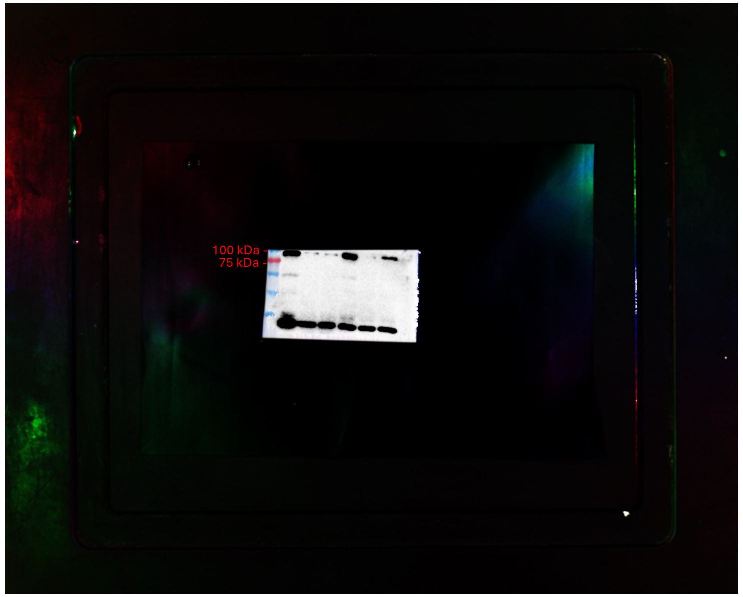

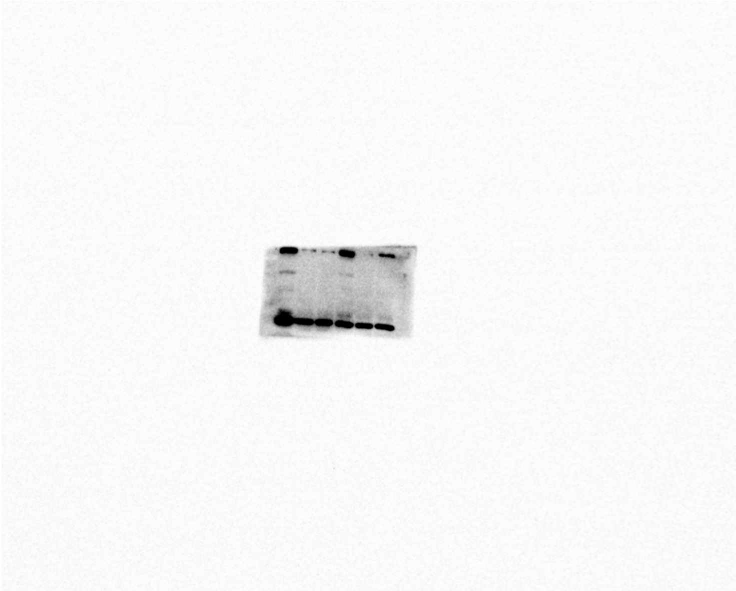

MFN2 ≈ mw 86 fig 3

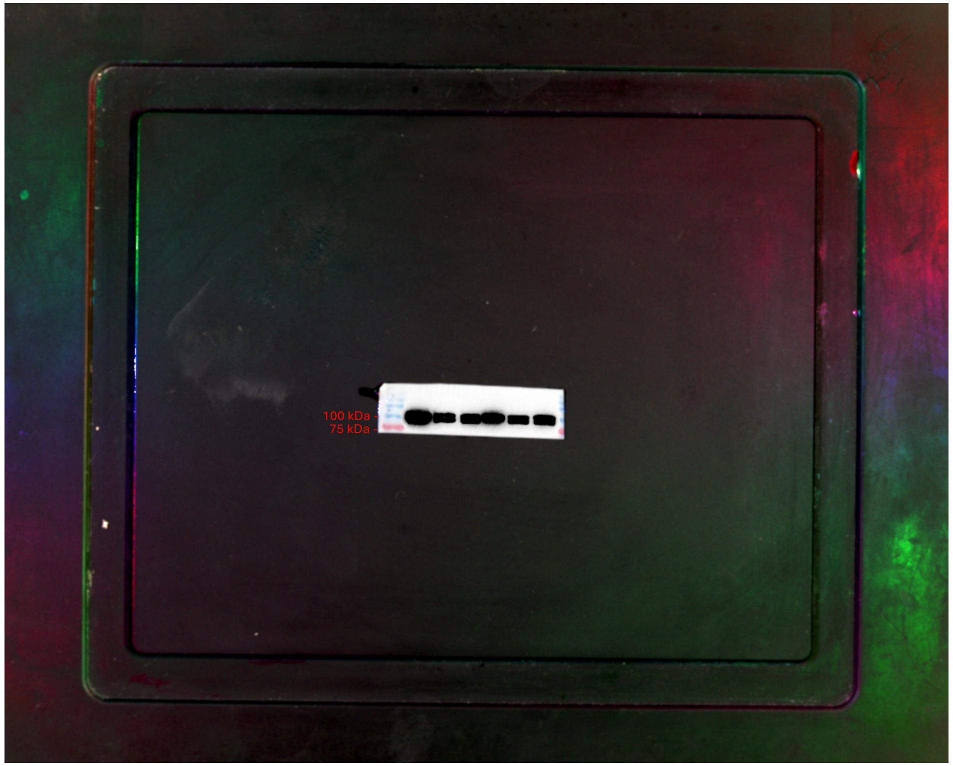

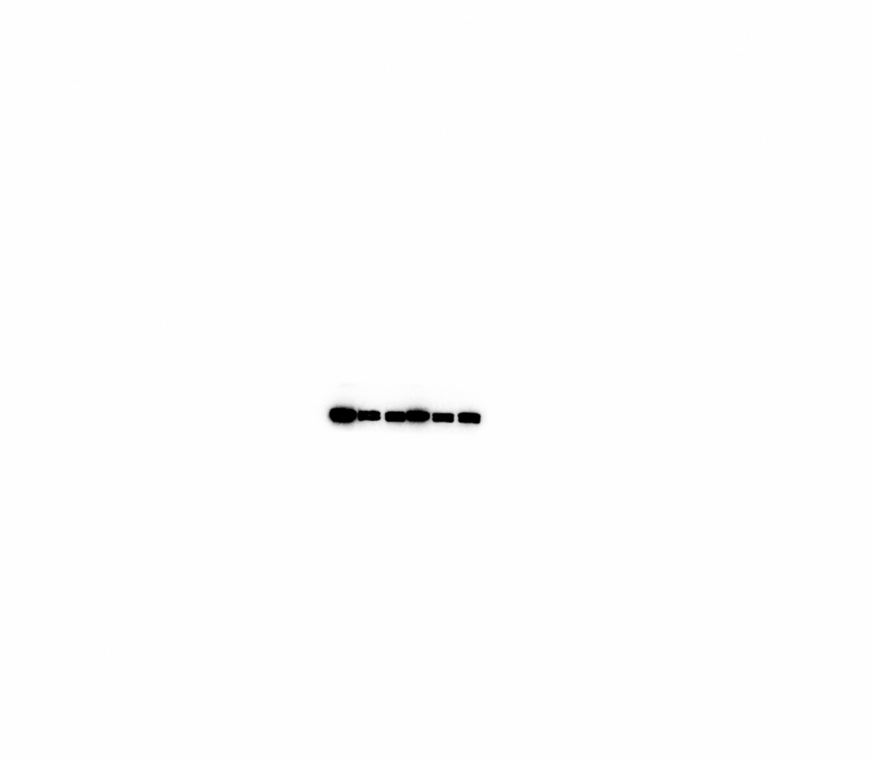

DRP1 ≈ mw 82 fig 3

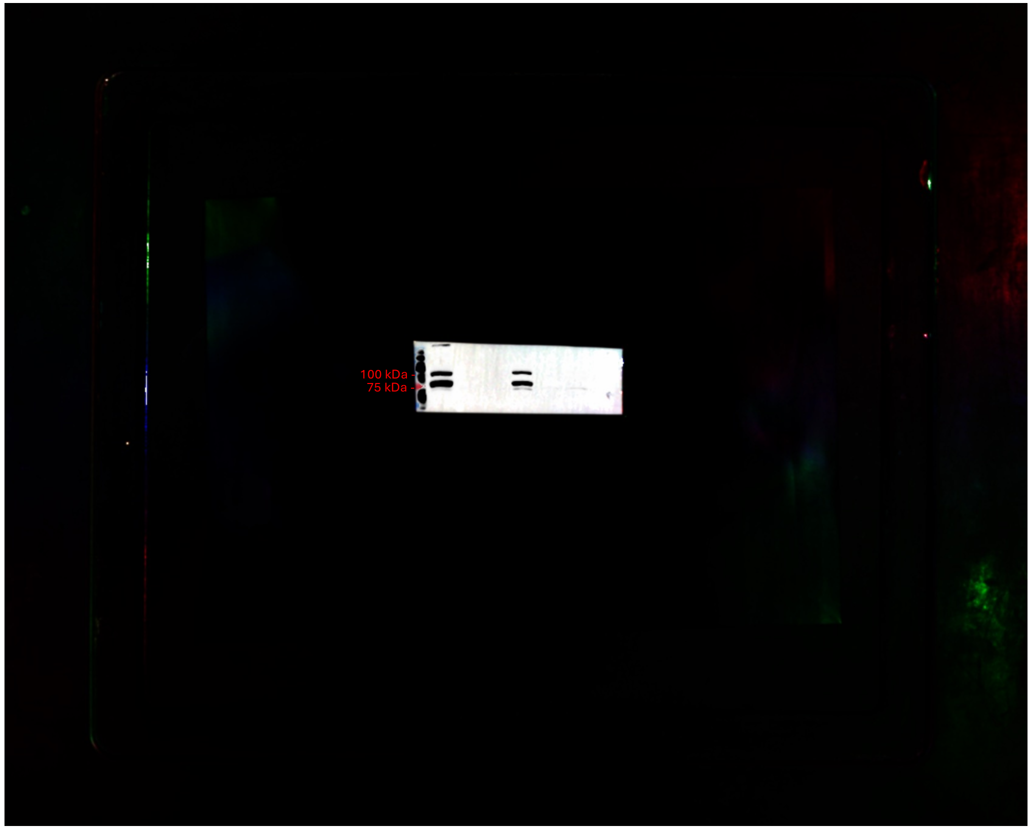

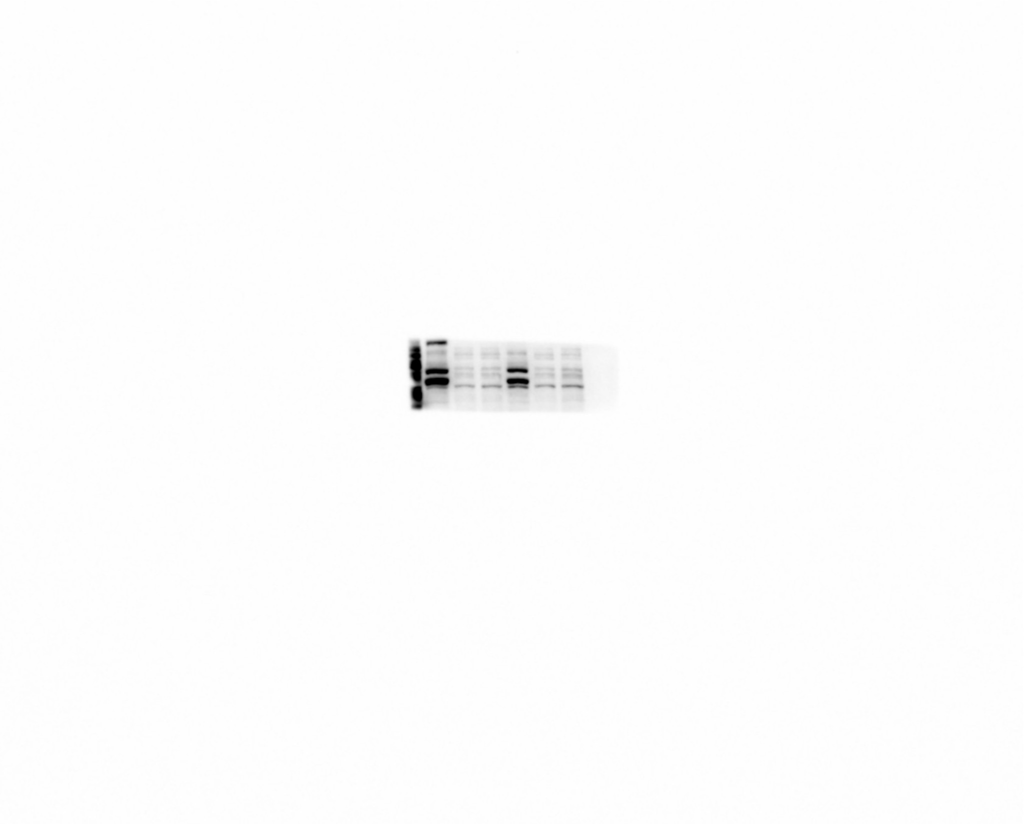

FIS1 ≈ mw 16 fig 3

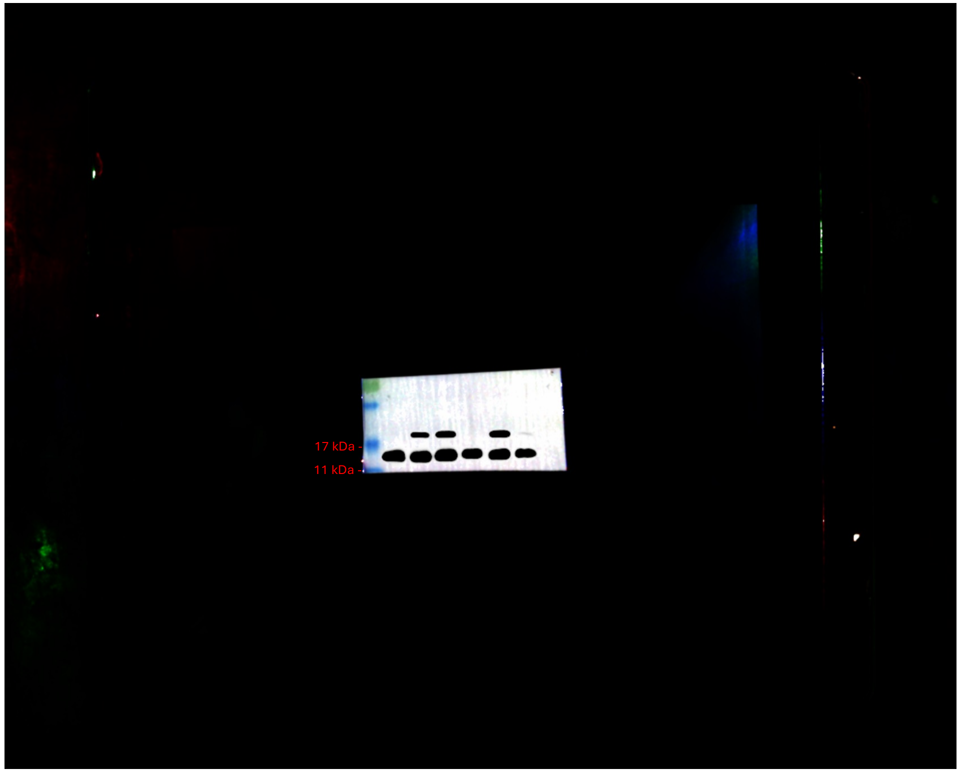

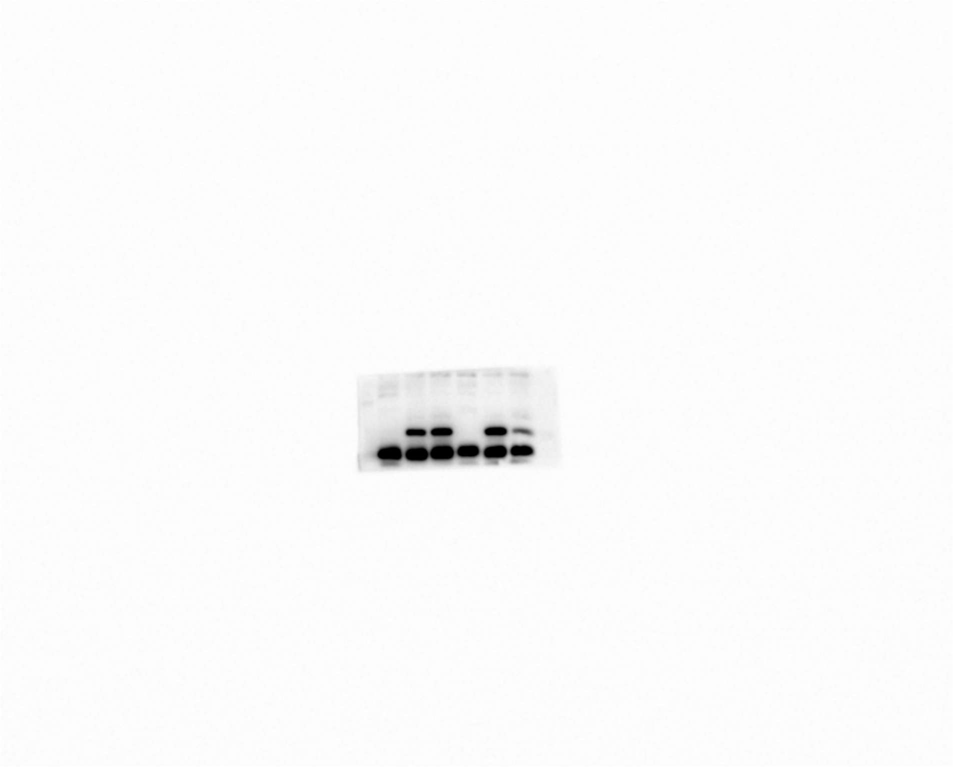

COX1 ≈ mw 37 fig 3

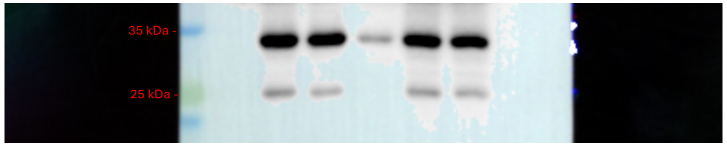

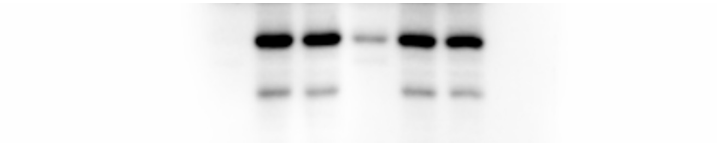

The acquisition was carried out using a selected region of interest (ROI) with the system configured for selective area exposure; consequently, the resulting image appears cropped. In this way also low signal can be acquired.

SDHA ≈ mw 70 fig 3

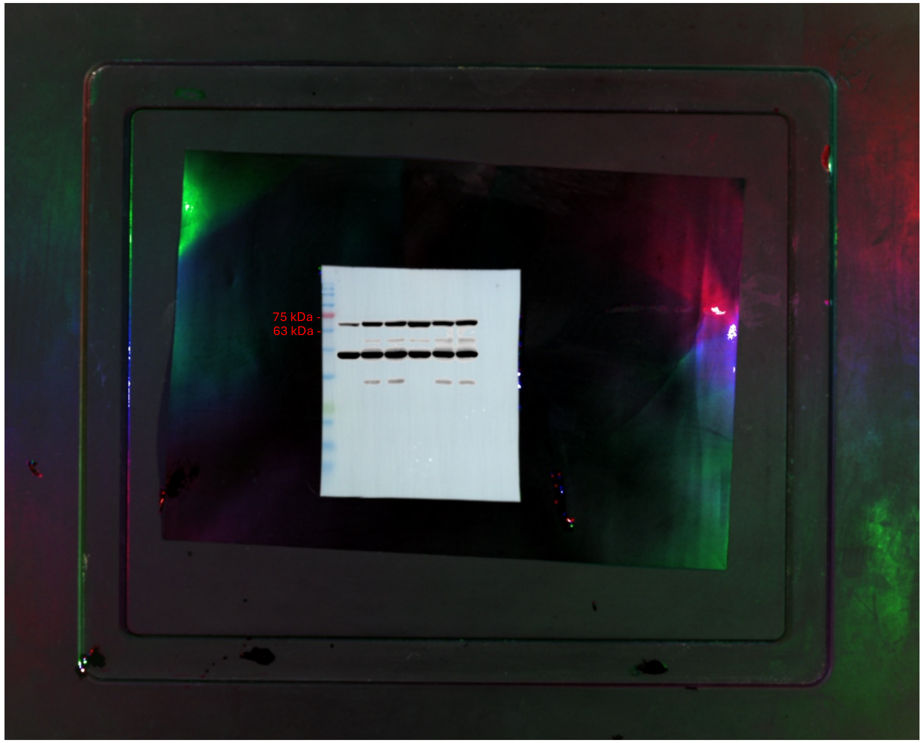

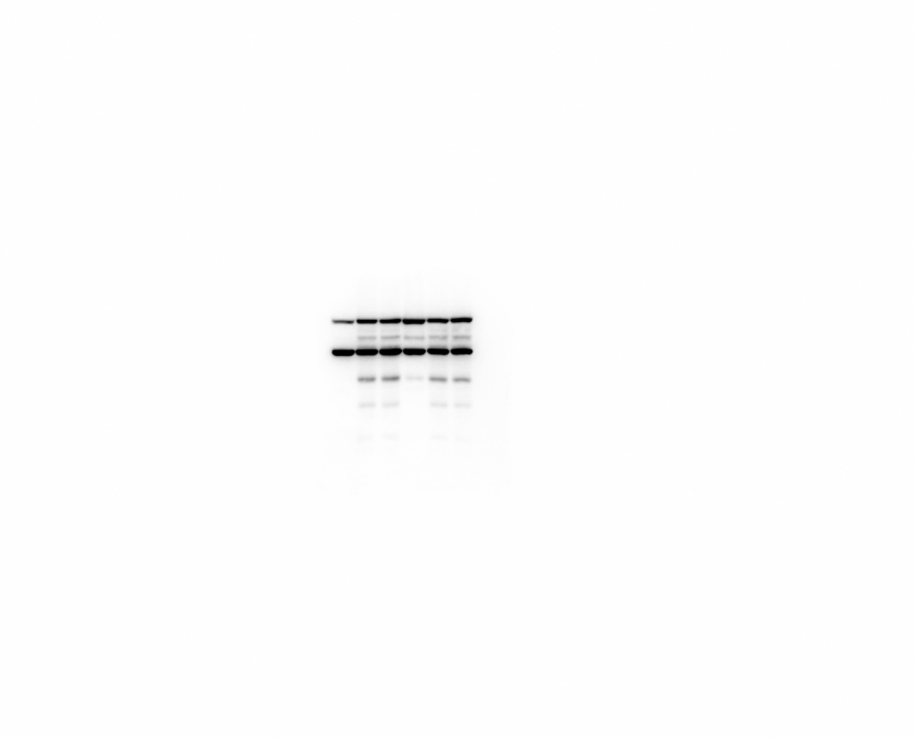

Multiple bands can be observed because the ab123545 MitoBiogenesis™ Western Blot Cocktail was used, allowing the simultaneous detection of SDHA, β-Actin, and COX1.

Beta Actin ≈ mw 42 fig 3

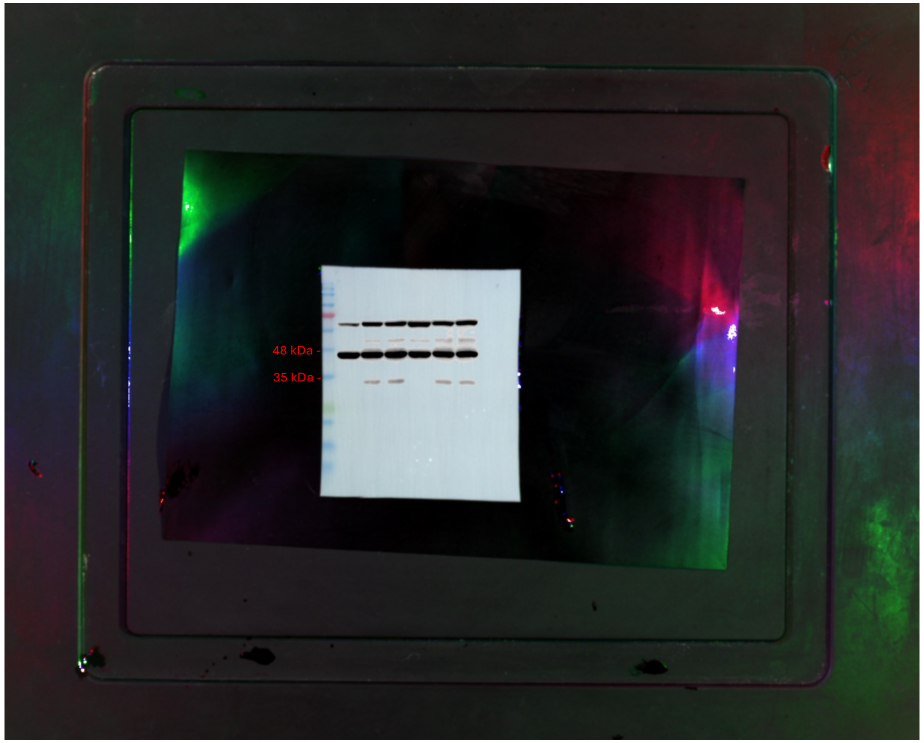

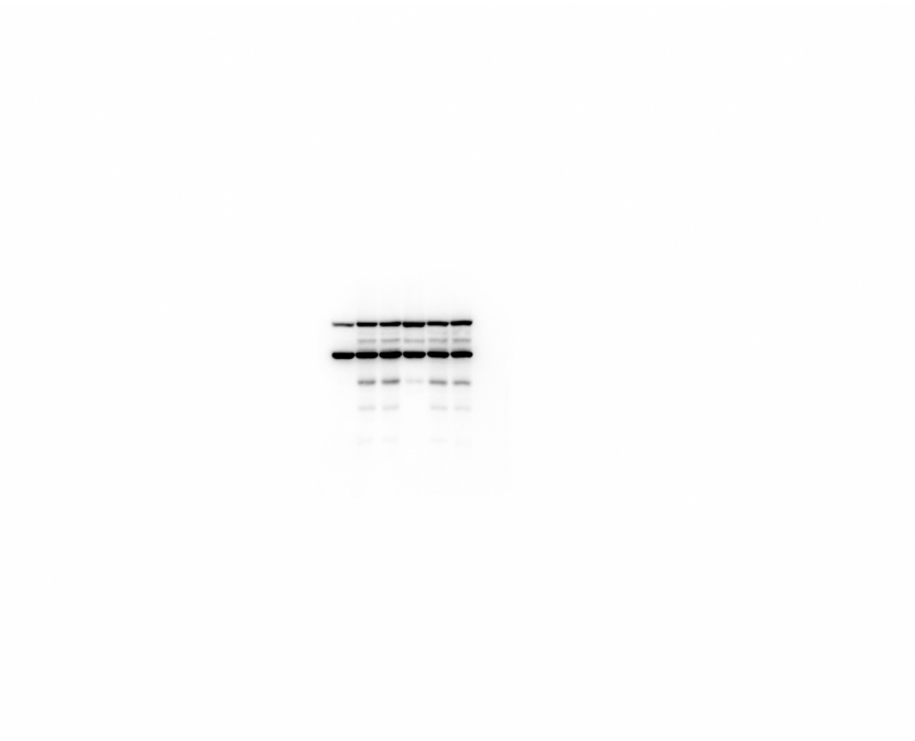

Multiple bands can be observed because the ab123545 MitoBiogenesis™ Western Blot Cocktail was used, allowing the simultaneous detection of SDHA, β-Actin, and COX1.

RB1≈ mw 110 fig 4

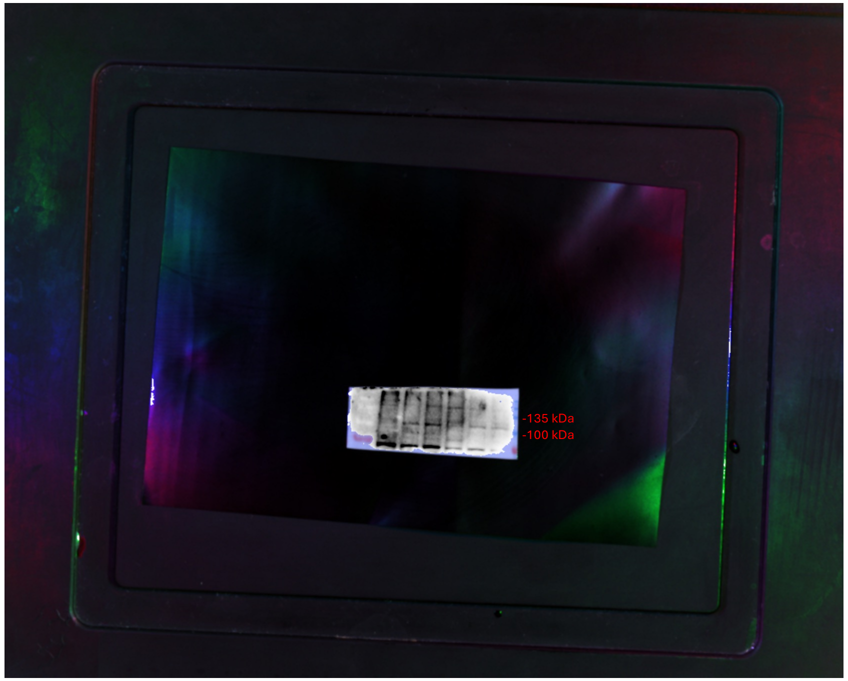

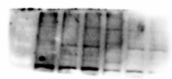

p53 ≈ mw 53 kDa fig 4

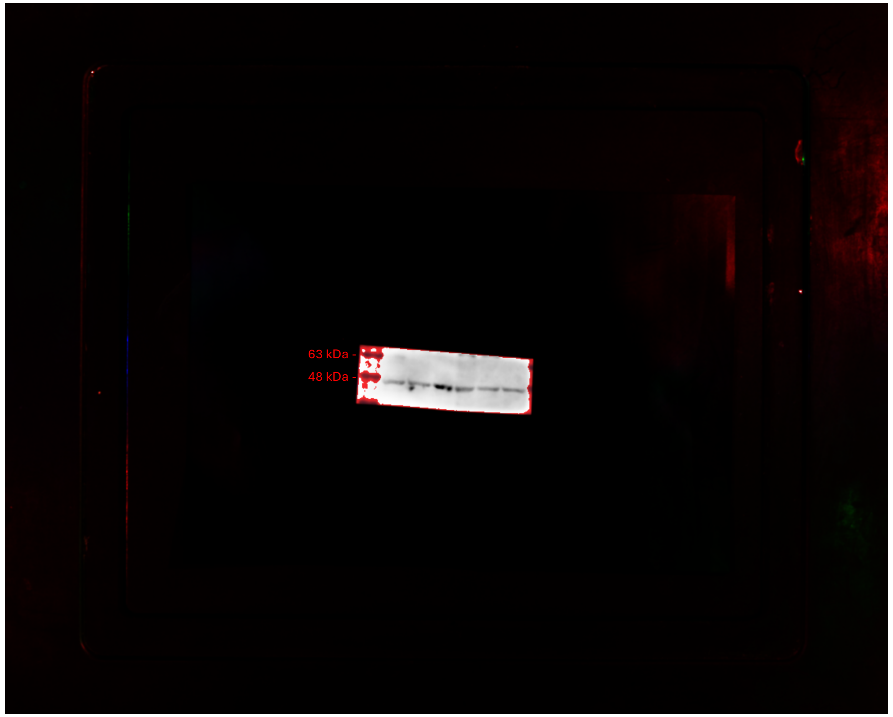

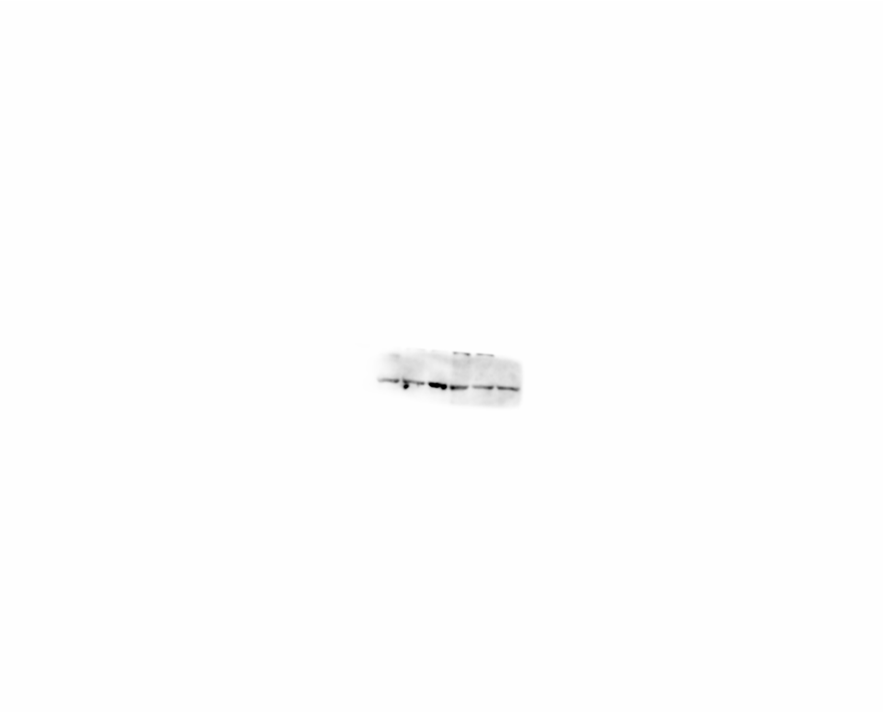

p21≈ mw 21 fig 4

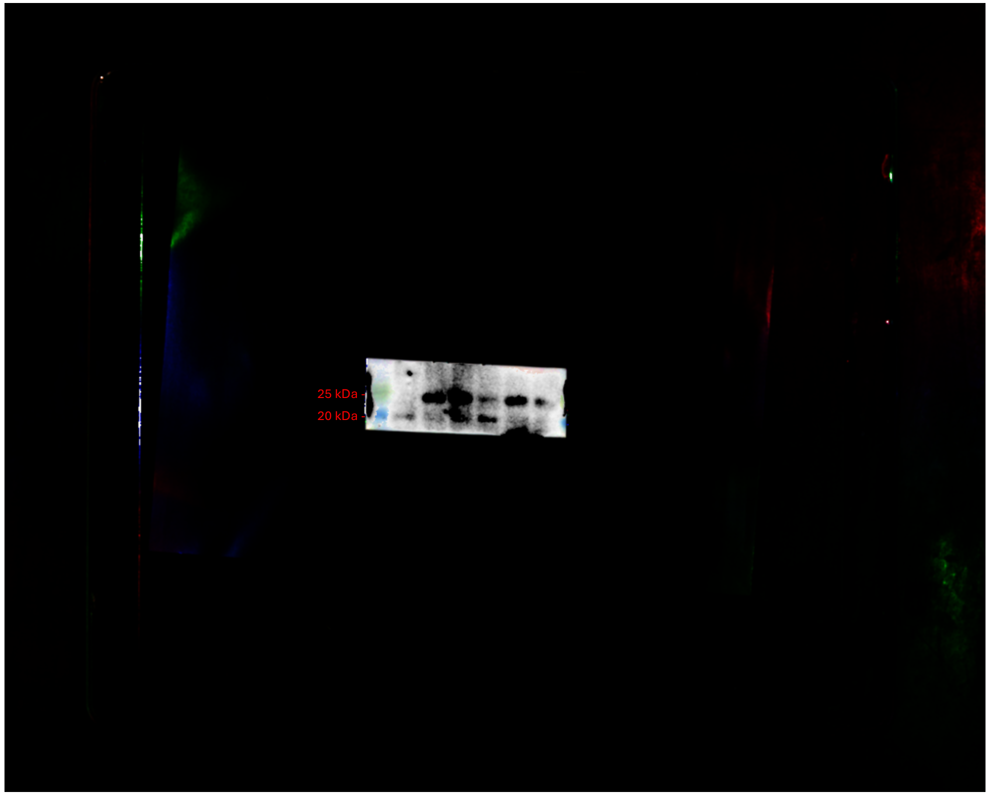

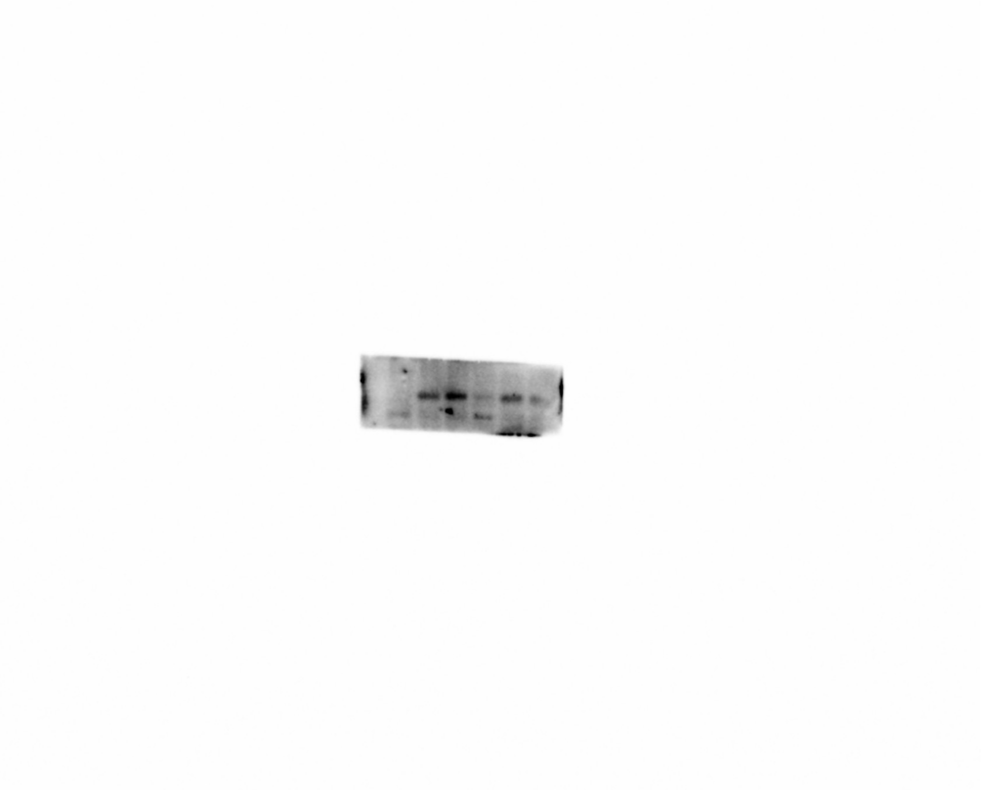

p16≈ mw 16/32 fig 4

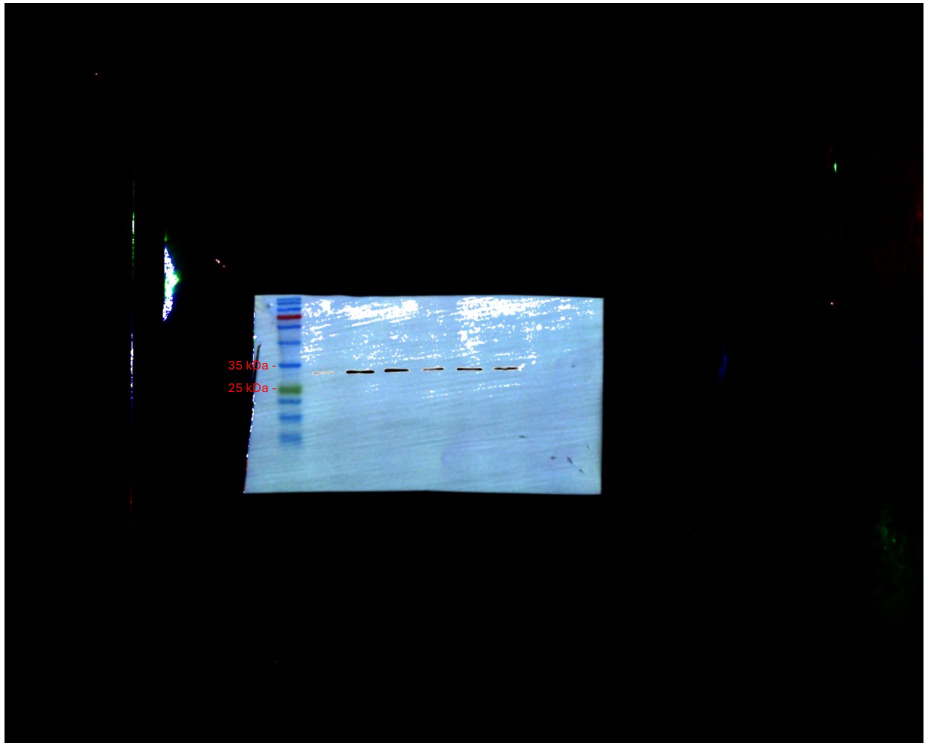

Beta Actin ≈ mw 42 fig 4

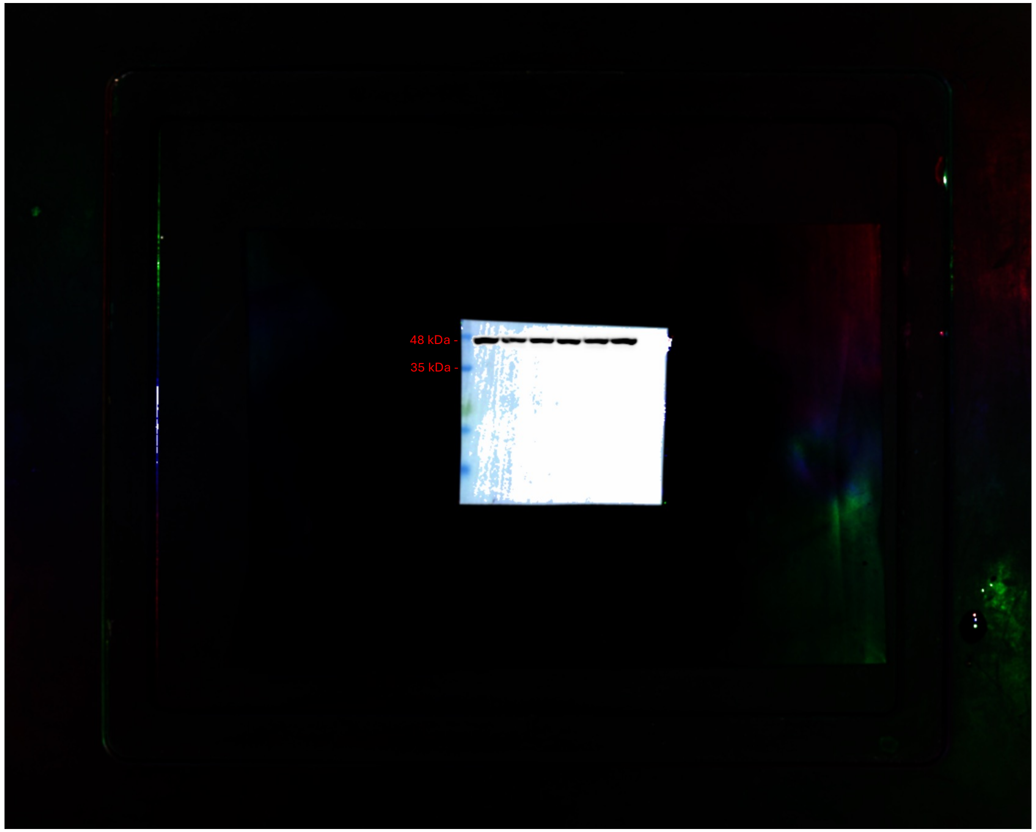

Supplement: Supplementary file 1 [file antioxidants-15-00372-s001.zip › antioxidants-4168788-supplementary.pdf]
